# Complete genome analysis of African swine fever virus responsible for outbreaks in domestic pigs in 2018 in Burundi and 2019 in Malawi

**DOI:** 10.1007/s11250-021-02877-y

**Published:** 2021-08-17

**Authors:** Jean N. Hakizimana, Jean B. Ntirandekura, Clara Yona, Lionel Nyabongo, Gladson Kamwendo, Julius L. C. Chulu, Désiré Ntakirutimana, Olivier Kamana, Hans Nauwynck, Gerald Misinzo

**Affiliations:** 1 SACIDS Africa Centre of Excellence for Infectious Diseases, SACIDS Foundation for One Health, Sokoine University of Agriculture, PO Box 3297, Morogoro, Tanzania; 2 Department of Veterinary Microbiology, Parasitology and Biotechnology, College of Veterinary Medicine and Biomedical Sciences, Sokoine University of Agriculture, PO Box 3019, Morogoro, Tanzania; 3 Department of Animal Health and Productions, University of Burundi, PO Box 1550, Bujumbura, Burundi; 4 Department of Biosciences, Solomon Mahlangu College of Science and Education, Sokoine University of Agriculture, PO Box 3038, Morogoro, Tanzania; 5 National Veterinary Laboratory of Burundi, PO Box 227, Bujumbura, Burundi; 6 Department of Animal Health and Livestock Development, Ministry of Agriculture, Irrigation and Water Development, PO Box 2096, Lilongwe, Malawi; 7 Department of Applied Research and Development and Foresight Incubation, National Industrial Research and Development Agency, PO Box 273, Kigali, Rwanda; 8 Laboratory of Virology, Faculty of Veterinary Medicine, Ghent University, Salisburylaan 133, 9820 Merelbeke, Belgium

**Keywords:** African swine fever virus, *Asfarviridae*, Burundi, Domestic pig, Malawi, Whole-genome sequencing

## Abstract

Several African swine fever (ASF) outbreaks in domestic pigs have been reported in Burundi and Malawi and whole-genome sequences of circulating outbreak viruses in these countries are limited. In the present study, complete genome sequences of ASF viruses (ASFV) that caused the 2018 outbreak in Burundi (BUR/18/Rutana) and the 2019 outbreak in Malawi (MAL/19/Karonga) were produced using Illumina next-generation sequencing (NGS) platform and compared with other previously described ASFV complete genomes. The complete nucleotide sequences of BUR/18/Rutana and MAL/19/Karonga were 176,564 and 183,325 base pairs long with GC content of 38.62 and 38.48%, respectively. The MAL/19/Karonga virus had a total of 186 open reading frames (ORFs) while the BUR/18/Rutana strain had 151 ORFs. After comparative genomic analysis, the MAL/19/Karonga virus showed greater than 99% nucleotide identity with other complete nucleotides sequences of p72 genotype II viruses previously described in Tanzania, Europe and Asia including the Georgia 2007/1 isolate. The Burundian ASFV BUR/18/Rutana exhibited 98.95 to 99.34% nucleotide identity with genotype X ASFV previously described in Kenya and in Democratic Republic of the Congo (DRC). The serotyping results classified the BUR/18/Rutana and MAL/19/Karonga ASFV strains in serogroups 7 and 8, respectively. The results of this study provide insight into the genetic structure and antigenic diversity of ASFV strains circulating in Burundi and Malawi. This is important in order to understand the transmission dynamics and genetic evolution of ASFV in eastern Africa, with an ultimate goal of designing an efficient risk management strategy against ASF transboundary spread.

## Introduction

The aetiology of Africa swine fever (ASF) is ASF virus (ASFV), a linear double-stranded DNA arbovirus with a genome size ranging between 170 and 194 kilobase pairs (kbp), and the only member of the genus *Asfivirus*, family *Asfarviridae* (Alonso et al. 2018). However, a potential new member of the *Asfarviridae* family designated as Abalone asfa-like virus (AbALV) has been recently reported (Matsuyama et al. 2020). The outcome of ASF infection in domestic pigs and Eurasian wild boars depends on virulence of causative ASFV and ranges from acute to chronic disease with mortality rates approaching 100% in naïve population (Karger et al. 2019; Pikalo et al. 2019). Due to its high mortality rate, unavailability of a commercial vaccine or effective treatment, and trade restriction of domestic pigs and pork products across countries, ASF is considered as the most serious threat to the global domestic pig industry (Costard et al. 2009; Couacy-Hymann 2019; Onzere et al. 2018). Transmission of ASF is through direct contact between infected and susceptible domestic pigs or wild boars, ingestion of contaminated pork products, contact with infected fomites, indirect transmission through carcasses in the habitat in the case of wild boars, or bites by infected soft ticks of the *Ornithodoros moubata* complex (Chenais et al. 2018; Penrith and Vosloo 2009). Soft ticks of the *O. moubata* complex act as vectors of the ASFV while in eastern and southern Africa, asymptomatically infected wild suids mainly warthogs (*Phacochoerus africanus*) play an important role as ASFV reservoirs (Jori et al. 2013). The ASFV infection of other wild suids species such as bush pig (*Potamochoerus larvatus*) and giant forest hogs (*Hylochoerus meinertzhageni*) has been previously reported but their role in the epidemiology of the virus is not well known (Penrith et al. 2019).

Domestic pigs and the pig farming systems in Africa, South of the Sahara, have been reported to play an important role in the ASFV transmission and spread (Mwiine et al. 2019; Yona et al. 2020) while the high stability of ASFV in pork products is cited to be the major factor of ASFV spread across long distances. For instance, the first escape of the virus from Africa to Portugal in 1957 and again in 1960 was associated to airplane waste with contaminated pork products that was used for pig feeding while contaminated ship waste was cited to be the origin of ASFV introduction in Georgia in 2007 (Rowlands et al. 2008). More than 33 countries of Africa, South of the Sahara, have reported ASF where the disease is endemic and ASFV is becoming more prevalent in European and Asian countries threatening global food and nutritional security (Ge et al. 2019; Penrith et al. 2019).

The ASFV genome varies in size between 170 and 194 kilobase pairs (kbp) with a conserved central region of about 125 kbp, in addition to the left variable region (LVR) of 38 to 47 kbp and the right variable region (RVR) of 13 to16 kbp (de Villiers et al. 2010). The variation of the genome lengths of different ASFV strains is caused by the gain or loss of members of the five different multigene families (MGF) of ASFV found in the LVR and the RVR, for instance, MGFs 100, 110, 300, 360 and 530/505 (Alonso et al. 2018). Previous studies have reported between 151 and 167 ORFs in ASFV genomes (de Villiers et al. 2010). However, an increasing number of studies have reported more than 167 ORFs in ASFV genomes especially the strains belonging to ASFV p72 genotype II, including seven Polish isolates, collected between 2016 and 2017 with 187 to 190 ORFs (Mazur-Panasiuk et al. 2019) and the ASFV strain Belgium/Etalle/wb/2018 detected in wild boar in Belgium in 2018 with 186 ORFs (Gilliaux et al. 2019). A study that analyzed 12 complete genomes of the ASFV strains collected in Sardinia, Italy, from 1978 to 2014 reported 231 ORFs in four isolates and 235 ORFs in eight ASFV isolates with 66 ORFs defined as uncharacterized (Torresi et al. 2020).

Based on partial nucleotide sequence analysis of the *B646L* gene that encodes for the major capsid protein p72, 24 (I–XXIV) ASFV genotypes have been identified and all of these have been reported to circulate in Africa, South of the Sahara (Achenbach et al. 2017; Lubisi et al. 2007; Quembo et al. 2018). Previous studies have reported ASFV p72 genotypes II, V, VIII and XII in Malawi while only ASFV p72 genotype X was reported in Burundi (Hakizimana et al. 2020a, b; Lubisi et al. 2005). Currently, only 3 complete and fully annotated ASFV strains belonging to p72 genotype X are available in the GenBank, including two strains from Kenya and one from Democratic Republic of the Congo (DRC) (Bisimwa et al. 2021; de Villiers et al. 2010). However, despite the endemic status of ASF in Burundi, no ASFV has been fully sequenced. In addition, there is no ASFV p72 genotype II strain from Malawi that has been subjected to complete genome sequencing. In this study, we report the complete genome sequences of ASFV p72 genotype X (BUR/18/Rutana) responsible for the 2018 ASF outbreak in Burundi and ASFV 72 genotype II (MAL/19/Karonga) that caused an outbreak during 2019 in Malawi.

## Materials and methods

### Sequencing of the ASFV complete genome

Collection of the samples used in this study and subsequent ASF confirmation and genotyping have been previously described (Hakizimana et al. 2020a, b). Viral DNA was extracted from tissue samples using the Quick-DNA™ Miniprep Plus Kit (Zymo Research Corporation, CA, USA), following the manufacturer’s instructions. Assessment of the integrity and quality of the extracted DNA was done through 1% agarose gel electrophoresis for 30 min running at 160 V with 0.5μL of sample DNA loaded. The starting genomic DNA for complete genome sequencing was quantified by picogreen method (Invitrogen, Catalog # P7589) using Victor 3 fluorometry (PerkinElmer Life and Analytical Sciences, Shelton, USA). Illumina NovaSeq6000 instrument with 2 × 150 bp configuration was used for sequencing and TruSeq Nano DNA Kit (Catalog # 20,015,964) was used for library preparation, according to the manufacturer’s protocol. Quality control of the prepared library was done by 2100 Bioanalyzer using a DNA 1000 chip (Agilent Technologies, USA) while the library quantification was performed using real-time polymerase chain reaction (qPCR) according to the Illumina qPCR Quantification Protocol Guide (Catalog # SY-930–1010). The libraries were subjected to sequencing to produce approximately 28 million paired-end reads (4 GB) per sample.

### Assembly and annotation of the ASFV genome

Adapter sequences and low-quality reads trimming were performed using Trim Galore version 0.6.4 (https://www.bioinformatics.babraham.ac.uk/projects/trim_galore/) with cutadapt version 2.8 and the quality Phred score cutoff was set to 30 with a minimum reads length of 75 nucleotides. The quality of the filtered sequence data was assessed using FastQC version 0.11.9 (Andrews 2010). The quality-filtered reads were de novo assembled using SPAdes version 3.13.1 (Bankevich et al. 2012) and Megahit version 1.2.9 (Li et al. 2015). The assembly contigs were mapped to the reference genome using Burrows-Wheeler Aligner (BWA) version 0.7.17 with maximum exact match (mem) option (Li 2013) and the QUAST program version 5.0.2 (Gurevich et al. 2013) was used to evaluate the quality of the assembly. The longest overlapping scaffolds were assembled to generate the ASFV complete genomes. The Genome Annotation Transfer Utility (GATU) software (Tcherepanov et al. 2006) was used for annotation of the assembled ASFV genomes using Georgia 2007/1 (GenBank accession number NC_044959.2) and Ken05/Tk1 (GenBank accession number NC_044945.1) as reference genomes. The basic local alignment search tool for nucleotide (BLASTN) version 2.11.0 + (Zhang et al. 2000) was used for pairwise nucleotide alignment and search for nucleotide identity at GenBank nucleotide database. Multiple sequence alignment was carried out using MAFFT program version 7.221 (Katoh and Standley 2013) and the evolutionary history was inferred using the maximum likelihood method with 1000 bootstrap replications and evolutionary distances were calculated using Kimura 2-parameter model (Kimura 1980) as implemented in MEGA X (Kumar et al. 2018).

## Results

### Characteristics of the complete genomes of Burundian and Malawian ASFV strains

**Fig. 1:**
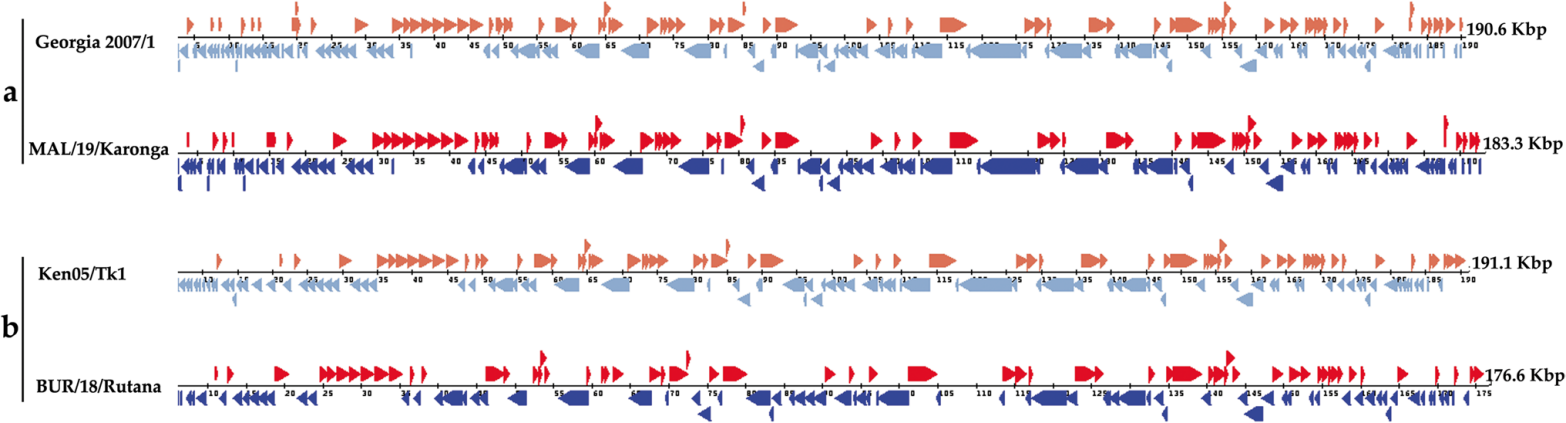
Graphical display of African swine fever virus open reading frames (ORFs) of MAL/19/Karonga (**a**) and BUR/18/Rutana (**b**.) automatically annotated by Genome Annotation Transfer Utility (GATU) using corresponding very closely related African swine fever virus reference genomes. The direction of arrows indicates the 5′ to 3′orientation of ORFs

Complete genome sequences of the ASFV strains responsible for the 2018 outbreak in Rutana region, South-eastern Burundi (BUR/18/Rutana), and the 2019 outbreak in Karonga district, northern Malawi (MAL/19/Karonga), were determined in this study. The strains BUR/18/Rutana and MAL/19/Karonga belong to ASFV p72 genotypes X and II, respectively, as previously described through partial genome amplification and sequencing targeting specific genomic regions (Hakizimana et al. 2020a, b). The complete genome assembly generated genomes of 176,564 bp for BUR/18/Rutana and 183,325 bp for MAL/19/Karonga with GC content of 38.62 and 38.48%, respectively. The MAL/19/Karonga strain had a total of 186 open reading frames (ORFs) while the BUR/18/Rutana strain had 151 ORFs as highlighted by the whole-genome alignment of homologous genes between the ASFV strains described in this study and the corresponding reference genomes (Fig. 1). For MAL/19/Karonga, a total of 44 multigene family (MGF) members were identified within the genome including MGF 100 (3 members), MGF 110 (10 members), MGF 300 (3 members), MGF 360 (18 members) and MGF 505 (10 members). Furthermore, 36 MGF members were identified within the genome of BUR/18/Rutana strain including MGF 100 (1 member), MGF 110 (8 members), MGF 300 (3 members), MGF 360 (16 members) and MGF 505 (8 members). The complete genome sequences generated in this study were submitted to GenBank and assigned accession numbers (MW856067 for BUR/18/Rutana and MW856068 for MAL/19/Karonga).

### Comparative genomic analysis

**Fig. 2:**
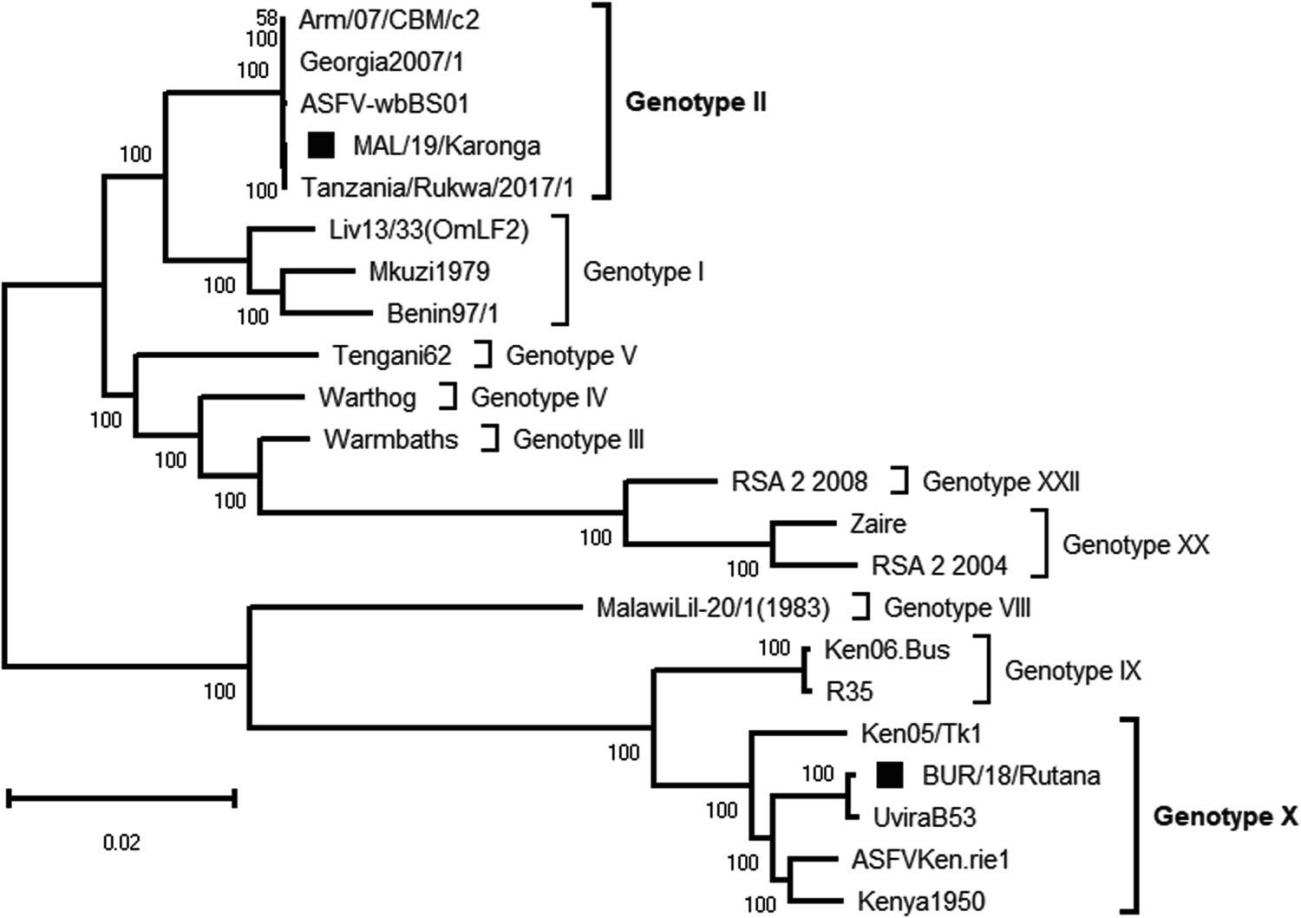
Maximum likelihood phylogenetic tree obtained after multiple sequence alignment of complete genomes of African swine fever virus strains from Africa and selected strains from Europe and Asia. The viruses described in this study are indicated by black squares and the scale bar indicates nucleotide substitution per site while the node values show percentage of bootstrap support. The analysis involved 22 nucleotide sequences with a total of 166,578 positions in the final dataset

Using complete genome sequences for BLASTN search at the GenBank, the MAL/19/Karonga virus was closely related to Tanzania/Rukwa/2017/1 (GenBank accession number LR813622) ASFV strain collected in South-western Tanzania from an infected domestic pig during an ASF outbreak in 2017 and belonging to ASFV p72 genotype II, with 99.97% nucleotide identity. The percentage of nucleotide identity was greater than 99% with other complete genomic sequences of ASFV belonging to p72 genotype II isolated in Europe and Asia including the Georgia 2007/1 isolate. On the other hand, the BUR/18/Rutana ASFV strain exhibited 99.34%, 99.08% and 98.95% nucleotide identity with the Uvira B53 (Bisimwa et al. 2021), Ken05/Tk1 (Bishop et al. 2015) and Kenya 1950 (GenBank accession number AY261360) ASFV p72 genotype X strains, respectively (Table 1). Phylogenetic reconstruction using complete genomes clustered the MAL/19/Karonga and BUR/18/Rutana viruses into ASFV genotypes II and X, respectively (Fig. 2). With a genome size of 183,325 bp, the MAL/19/Karonga strain was 139 bp longer than the Tanzania/Rukwa/2017/1 (183,186 bp) and about 6 to 7 kbp shorter than some ASFV p72 genotype II isolates available in the GenBank nucleotide database, for instance Georgia 2007/1 (190,584 bp), Arm/07/CBM/c2 (190,145 bp) and ASFV-wbBS01 (189,394 bp). Furthermore, the BUR/18/Rutana strain with the genome length of 176,564 bp was about 4 to 17 kbp shorter than the Uvira B53 (180,916 bp), Ken05/Tk1 (191,058 bp) and Kenya 1950 (193,886 bp) ASFV p72 genotype X genomes. The difference in genome length is due to differences within some genes and MGF members. For instance, among the 186 ORFs identified in MAL/19/Karonga, 151 ORFs had 100% nucleotide identity with their homologues in the reference genome while the remaining 35 ORFs were polymorphic with nucleotide identity with the reference genome varying from 60 to 99.9%. In addition, four ORFs (MGF 360-1Lb, ASFV G ACD 00,120, MGF 110-7L and MGF 110–10-L—MGF110-14L fusion) present in ASFV reference genome were below the 60% nucleotide identity threshold and considered absent in the MAL/19/Karonga virus. Among the 151 ORFs identified in BUR/18/Rutana virus, only 35 ORFs had 100% nucleotide identity with their homologues in Ken05/Tk1 reference genome while the remaining ones had between 60 and 99.9% identity. Besides, 10 MGF members (MGF 110-7L, MGF 110-8L, MGF 100-1R, MGF 110-9L, MGF 110-11L (FRAG-2), MGF 110-13L-14L, MGF 360-12L, MGF 360-15R, MGF 100-3L, MGF 360-18R) present in the Ken05/Tk1 reference genome had a nucleotide similarity below 60% compared to their homologues in BUR/18/Rutana virus.

**Table 1:** Publicly available complete genome sequences of African swine fever virus strains from Africa and selected strains from Europe and Asia used for comparative genomic analysis in this study

Name of the strain	GenBank accession number	Country of origin	Year of collection	p72 genotype	Length (bp)	Percentage of identity with MAL/19/Karonga (%)	Percentage of identity with BUR/18/Rutana (%)	Host species	Reference
Mkuzi1979	AY261362	South Africa	1979	I	192,714	97.95	93.35	Tick	Unpublished
Benin 97/1	NC_044956	Benin	1997	I	182,284	97.91	93.01	Domestic pig	(Chapman et al., 2008)
Liv13/33(OmLF2)	MN913970	Zambia	1983	I	188,277	98.29	93.29	Tick	(Chastagner et al. 2020)
MAL/19/Karonga	MW856068	Malawi	2019	II	183,325	100	93.42	Domestic pig	This study
Arm/07/CBM/c2	LR812933	Armenia	2007	II	190,145	99.95	93.45	Domestic pig	Unpublished
Georgia 2007/1	NC_044959.2	Georgia	2007	II	190,584	99.95	93.45	Domestic pig	(Chapman et al. 2011)
ASFV-wbBS01	MK645909	China	2018	II	189,394	99.90	93.44	Wild boar	Unpublished
Tanzania/Rukwa/2017/1	LR813622	Tanzania	2017	II	183,186	99.97	93.44	Domestic pig	(Njau et al. 2021)
Warmbaths	AY261365	South Africa	1987	III	190,773	96.83	93.45	Tick	Unpublished
Warthog	AY261366	Namibia	1980	IV	186,528	97.50	93.11	Warthog	Unpublished
Tengani62	AY261364	Malawi	1962	V	185,689	96.72	93.34	Domestic pig	Unpublished
MalawiLil-20/1(1983)	AY261361	Malawi	1983	VIII	187,612	94.62	93.42	Tick	Unpublished
Ken06.Bus	NC_044946	Kenya	2006	IX	184,368	92.78	97.71	Domestic pig	(Bishop et al. 2015)
R35	MH025920	Uganda	2015	IX	188,629	92.79	97.71	Domestic pig	(Masembe et al. 2018)
BUR/18/Rutana	MW856067	Burundi	2018	X	176,564	93.42	100	Domestic pig	This study
Ken05/Tk1	NC_044945	Kenya	2005	X	191,058	93.74	99.08	Tick	(Bishop et al. 2015)
ASFV Ken.rie1	LR899131	Kenya	2019	X	189,950	93.49	98.98	Tick	Unpublished
Uvira B53	MT956648	DRC	2019	X	180,916	92.26	99.34	Domestic pig	(Bisimwa et al. 2021)
Kenya 1950	AY261360	Kenya	1950	X	193,886	93.64	98.95	Domestic pig	Unpublished
Zaire	MN630494.2	DRC	1977	XX	184,820	96.68	93.77	Domestic pig	(Ndlovu et al. 2020a)
RSA_2_2004	MN641877.2	South Africa	2004	XX	189,903	95.27	92.22	Wild boar	(Ndlovu et al. 2020a)
RSA_2_2008	MN336500.3	South Africa	2008	XXII	190,066	94.17	90.50	Tick	(Ndlovu et al. 2020b)

*DRC*, Democratic Republic of the Congo

### Determination of the serogroups of Burundian and Malawian ASFV strains based on EP402R (CD2v) gene sequences

**Fig. 3:**
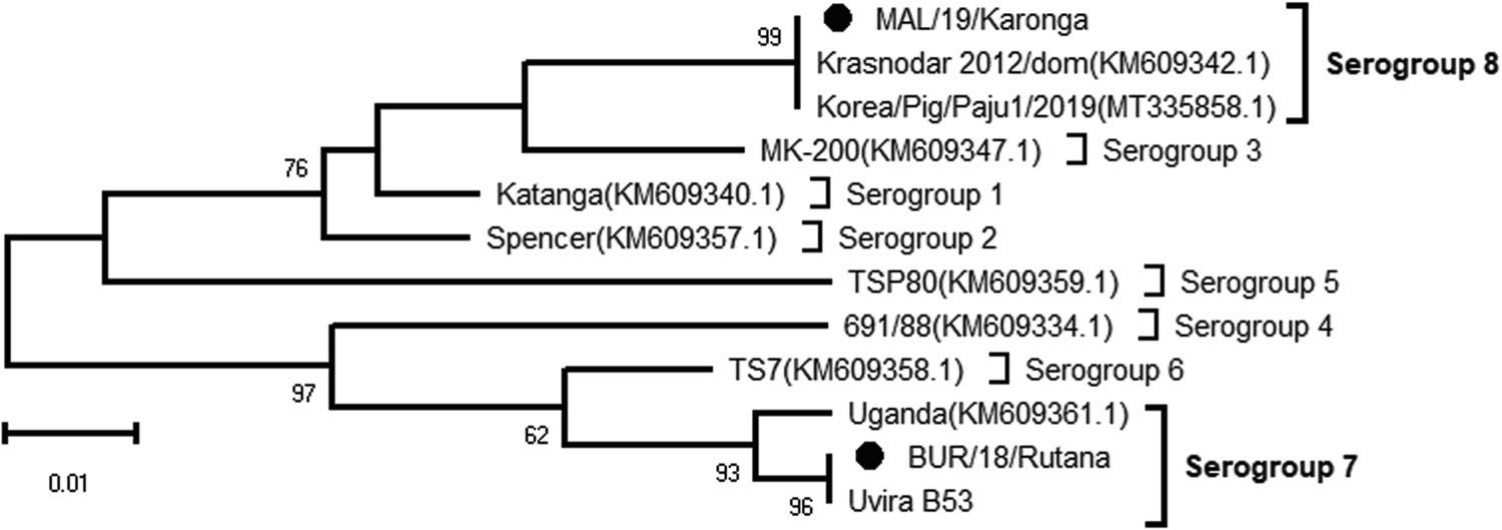
A maximum likelihood phylogenetic tree of the ASFV *EP402R* (CD2v) gene indicating serogroups of selected ASFV strains. The strains responsible for the 2018 and 2019 ASF outbreak in Burundi and in Malawi are indicated by a black dot. The scale bar indicates the number of substitutions per site

In order to classify the ASFV strains described in this study among the eight previously determined serogroups based on the ASFV hemadsorption inhibition (HAI) properties, we compared sequences of the *EP402R* gene that encodes the CD2v major ASFV antigen protein between them and selected isolates representing each serogroup retrieved from GenBank. A high nucleotide sequence variation was observed among the compared sequences and the serotyping results classified the BUR/18/Rutana and MAL/19/Karonga ASFV viruses in serogroups 7 and 8, respectively (Fig. 3). The Burundian ASFV strain grouped together with two strains belonging to serogroup 7 previously described, for instance the Uvira B53 ASFV strain collected during an ASF outbreak in South Kivu province of the Democratic Republic of the Congo (DRC) in 2019 and the Uganda ASFV strain (Bisimwa et al. 2021; Malogolovkin et al. 2015a, b), whereas the MAL/19/Karonga ASFV strain clustered together with strains belonging to ASFV serogroup 8 previously described in Europe and Asia.

## Discussion

The limited knowledge of the genetic variation of the ASFV has hindered the development of effective control and prevention strategies, including vaccine, diagnostic test and antiviral treatment development (Arabyan et al. 2019; Bao et al. 2021; Torresi et al. 2020; Urbano et al. 2021). Partial nucleotide sequencing of specific ASFV genomic regions is conventionally used to determine ASFV genotypes and to discriminate closely related ASFV strains. However, in order to obtain adequate information on transmission dynamics, genetic variation and molecular evolution of different ASFV strains, complete genome sequencing is required. To date, despite the regular reports of the ASFV p72 genotype II in different countries of eastern and southern Africa, only one fully annotated complete genome of the genotype II from those countries is publicly available, for instance the Tanzania/Rukwa/2017/1 collected in South-western Tanzania from an infected domestic pig during an outbreak in 2017 (Njau et al. 2021). There is no ASFV p72 genotype II strain from Malawi that has been subjected to complete genome sequencing and no ASFV strain from Burundi that has been fully sequenced. In the present study, complete genome sequences of the ASFV p72 genotype X responsible for the 2018 outbreak in Burundi and genotype II virus that caused the 2019 ASF outbreak in Malawi were generated using Illumina NGS technology. The complete genome sequences generated in this study were closely related to ASFV strains previously described, available in the GenBank database, belonging to ASFV p72 genotype X for the BUR/18/Rutana strain from Burundi and to genotype II for the MAL/19/Karonga strain from Malawi. Besides, serotyping results classified the BUR/18/Rutana and MAL/19/Karonga ASFV strains into ASFV serogroups 7 and 8, respectively.

The Burundian ASFV strain was more closely related to Uvira B53 ASFV strain collected during an ASF outbreak in in South Kivu province of the DRC (Bisimwa et al. 2021), with 99.34% nucleotides identity. These findings are in agreement with the results of studies using partial nucleotide sequencing where relatedness between those two ASFV strains were reported (Bisimwa et al. 2020; Hakizimana et al. 2020b) highlighting the possibility of transboundary spread of genotype X viruses between Burundi and DRC, as previously speculated. Furthermore, the Malawian ASFV strain described in this study was more closely related to the Tanzania/Rukwa/2017/1 ASFV strain collected in South-western Tanzania from an infected domestic pig during an ASF outbreak in 2017 (Njau et al. 2021), with 99.97% nucleotide identity. The high nucleotide similarity between ASFV p72 genotype II strains circulating in Malawi and Tanzania has been previously reported by studies using partial nucleotide sequencing suggesting a common source and transboundary spread of ASFV between these two countries (Hakizimana et al. 2020a; Misinzo et al. 2012). In addition, the Malawian ASFV strain had more than 99% nucleotides identity with ASFV p72 genotype II viruses previously described in Europe and Asia suggesting a possible common ancestor of these ASFV strains as previously speculated (Hakizimana et al. 2020a; Misinzo et al. 2012; Quembo et al. 2018; Rowlands et al. 2008).

Comparative genomic analysis revealed genetic variation in the ASFV strains described in this study compared to ASFV genomes previously described available in the GenBank. For instance, the *DP96R* gene reported as absent in the Uvira B53 ASFV strain was present in BUR/18/Rutana and MAL/19/Karonga strains with 93.6% and 100% nucleotide identity with the Ken05/Tk1 ASFV p72 genotype X and Georgia 2007/1 ASFV p72 genotype II reference genomes, respectively. The *DP96R* gene encodes the UK protein potentially involved in determining the ASFV virulence in domestic pigs (Zsak et al. 1998) and its presence in BUR/18/Rutana and MAL/19/Karonga ASFV strains may explain the high virulence of these strains as evidenced by high mortality rate during the 2018 and 2019 ASF outbreaks in Rutana region of Burundi and Karonga district in northern Malawi, as previously described (Hakizimana et al. 2020a, b). In addition, the *K196R* and the *B119L (9GL)* genes encoding the thymidine kinase and sulfhydryl oxidase enzymes, respectively, also described as the factors of virulence for ASFV (Rodríguez et al. 2015) were present in BUR/18/Rutana and MAL/19/Karonga ASFV strains.

Previous studies have reported important genetic variation within the members of the MGFs located at the both ends of the ASFV genome resulting in difference of the genome size of different ASFV strains (Torresi et al. 2020; Urbano et al. 2021). In the present study, several single-nucleotide polymorphisms (SNPs) and complete ORF deletion were observed within different MGF members. For instance, four MGF members (MGF 100-1R, MGF 110-7L, MGF 110-8L and MGF 110-9L) absent in the BUR/18/Rutana strains were also missing in the Uvira B53 strains as previously reported (Bisimwa et al. 2021). The MGF 360-1Lb gene was truncated in the MAL/19/Karonga strain and the same observation was reported in China/2018/AnhuiXCGQ ASFV strain collected during an ASF outbreak in domestic pigs in Anhui province of China in September 2018 (Bao et al. 2019). In addition, a deletion of almost all members of the MGF 110 were reported in the Estonia 2014 ASFV strain (Zani et al. 2018). The impact of these genetic variations on the phenotypes of the ASFV strains described in this study is subject to further investigations.

The protein pEP402R, a homologue of the T-lymphocyte surface antigen CD2, encoded by the *EP402R* gene is located in the lipoprotein membrane of the outer viral envelope and plays an important role in the adhesion of erythrocytes to infected cells (hemadsorption) and the binding of the ASFV particles to host erythrocytes during infection (Alejo et al. 2018; Dixon et al. 2019). This gene has been used to define eight viral antigenic types called serogroups (Malogolovkin and Kolbasov 2019). The results of the present study showed that the BUR/18/Rutana and MAL/19/Karonga ASFV strains may share the hemadsorption properties with ASFV strains belonging to serogroups 7 and 8, respectively (Fig. 3). It has been reported that the ASFV isolates classified into the same serotype show cross-protection responses from challenge during the vaccine development experiments (Malogolovkin et al. 2015a, b; Sánchez et al. 2019). Thus, the determination of the ASFV serogroups was suggested as a perfect tool for discriminating ASFV strains with different virulence and prediction of the efficacy of a specific ASFV vaccine (Burmakina et al. 2016). Recently, genetic signatures specific to each ASFV serotype have been described with the potential of elucidating more on the genetic and antigenic diversity of the ASFV (Malogolovkin et al. 2020; Urbano et al. 2021). Interestingly, the ASFV strains described in this study had the PPPKPC amino acid sequences repeated 4 and 3 times in the BUR/18/Rutana and MAL/19/Karonga ASFV strains, respectively. Similar tandem amino acid repeat sequences within the *EP402R* (CD2v) gene were reported in the Uvira B53 ASFV strain (Bisimwa et al. 2021).

In conclusion, the results of this study provided important insight into the genetic structure of the ASFV p72 genotype X responsible for the 2018 outbreak in Burundi and genotype II virus that caused the 2019 ASF outbreak in Malawi. Additionally, the strains BUR/18/Rutana and MAL/19/Karonga were classified into ASFV serogroups 7 and 8, respectively. These results will serve as backbone for possible future investigations concerning molecular evolution, transmission dynamics, diagnostic improvement and control strategies for ASFV.

## Data Availability

The nucleotide sequences generated in the present study were submitted to the NCBI GenBank with accession numbers MW856067 and MW856068.

## Declarations

The samples used in this study were collected from naturally dead domestic pigs according to common veterinary practice as part of routine veterinary investigation in Burundi and Malawi. In Burundi, sample collection was done according to the Burundian Animal Health Law (Law No. 1/28 of 24th December 2009) and the Law No. 1/06 of 21st March 2011 related to the practice of veterinary medicine in Burundi while in Malawi, the Control and Diseases of Animals Act (CAP 66:02 of 1967) and the rule 6 of the Swine Fever Rules G.N. 209/1968 were followed. Oral consent was obtained from the domestic pig owners before sampling of their dead domestic pigs and documented in the Veterinary Officer registry.

### Disclaimer

The funder had no role in study design, data collection and analysis, decision to publish and in the preparation of this manuscript.
